# Supplementary material for: Presentation and Clinical Course of Leptospirosis in a Referral Hospital in Far North Queensland, Tropical Australia
Source: Pathogens. 2025 Jun 28;14(7):643. doi: 10.3390/pathogens14070643 (PMC12300820; doi:10.3390/pathogens14070643)

Supplementary Table S1. Serovars in the *Leptospira* microscopic agglutination titre panel

**Species**	**Serogroup**	**Serovar**
Leptospira borgpetersenii	Ballum	Arborea
Leptospira interrogans	Australis	Australis
Leptospira interrogans	Bataviae	Bataviae
Leptospira kirschneri	Autumnalis	Bulgarica
Leptospira interrogans	Canicola	Canicola
Leptospira weilii	Celledoni	Celledoni
Leptospira interrogans	Icterohaemorrhagiae	Copenhageni
Leptospira kirschneri	Cynopteri	Cynopteri
Leptospira interrogans	Djasiman	Djasiman
Leptospira kirschneri	Grippotyphosa	Grippotyphosa
Leptospira borgpetersenii	Sejroe	Hardjo (subtype Hardjobovis)
Leptospira borgpetersenii	Javanica	Javanica
Leptospira interrogans	Hebdomadis	Kremastos
Leptospira noguchii	Panama	Panama
Leptospira interrogans	Pomona	Pomona
Leptospira interrogans	Pyrogenes	Robinsoni
Leptospira santarosai	Shermani	Shermani
Leptospira interrogans	Mini	Szwajizak
Leptospira borgpetersenii	Tarassovi	Tarassovi
Leptospira weilii	Tarassovi	Topaz
Leptospira interrogans	Pyrogenes	Zanoni

Supplementary Table S2: Definitions used for comorbidities in the cohort

**Comorbidity**		**Definition used**
If documented in patient's medical history recorded by the admitting doctor (categorical)	Diabetes mellitus	Documented in history, HbA1c > 6.5%, or on diabetic treatment
	Chronic Cardiac Failure	Documented in history, on heart failure therapy, or an echocardiogram demonstrating LVEF< 50%)
	Ischaemic heart disease	Documented in history as having a myocardial infarction or coronary artery bypass graft
	Chronic kidney disease	Documented in history or if baseline eGFR <90, CKD2 (60-90), CKD3a (45-59), 3b (30-44), 4 (15-29), 5 (<15/dialysis)
	Chronic lung disease	Documented in history; included interstitial lung disease, chronic obstructive pulmonary disease, bronchiectasis, cystic fibrosis, asthma or any other disease requiring therapy
	Chronic liver disease	Documented as having cirrhosis
	Active malignancy	Documented in history
	Autoimmune condition	Documented in history - any condition documented with – or without – immunomodulatory therapy
	Immunosuppression	If patient was taking any medications to suppress their immune system including regular corticosteroids, other immunosuppressants or immunomodulatory therapy
	Hazardous alcohol intake	>10 standard drinks per week or >4 standard drinks in one day at least once/month
	Smoker	Smoking tobacco on a regular basis

HbA1c: glycosylated haemoglobin; LVEF: left ventricular ejection fraction; eGFR: estimated glomerular filtration rate; CKD: chronic kidney disease.

Supplementary Table S3. Potential occupational or environmental exposure in the 81 individuals in the cohort in whom one could be determined.

**Occupational exposure**	**n (%)**
Freshwater swimming	16 (20)
Banana farmer	15 (19)
Cattle or another type of farmer	14 (17)
Other freshwater exposure	9 (11)
Camping	6 (7)
Domestic rodent infestation	6 (7)
Gardening	5 (6)
Other fruit picking	4 (5)
Forest worker	3 (4)
Veterinarian	1 (1)
Construction worker	1 (1)
Floodwater exposure	1 (1)
Total	81

Supplementary Table S4. Serovars of the 59 patients in whom a serovar was identified.

Serovar	n (%)
Zanoni	21 (36%)
Australis	12 (20%)
Robinsoni	6 (10%)
Arborea	5 (8%)
Kremastos	3 (5%)
Copenhageni	3 (5%)
Topaz	2 (3%)
Bulgarica	1 (2%)
Celledoni	1 (2%)
Cynopteri	1 (2%)
Hardjo	1 (2%)
Javanica	1 (2%)
Pomona	1 (2%)
Szwajizak	1 (2%)
Total	59

Supplementary Figure S1. Month of presentation of the patients with laboratory confirmed leptospirosis January 2015 to June 2024 (the local wet season runs from December to April, inclusive).

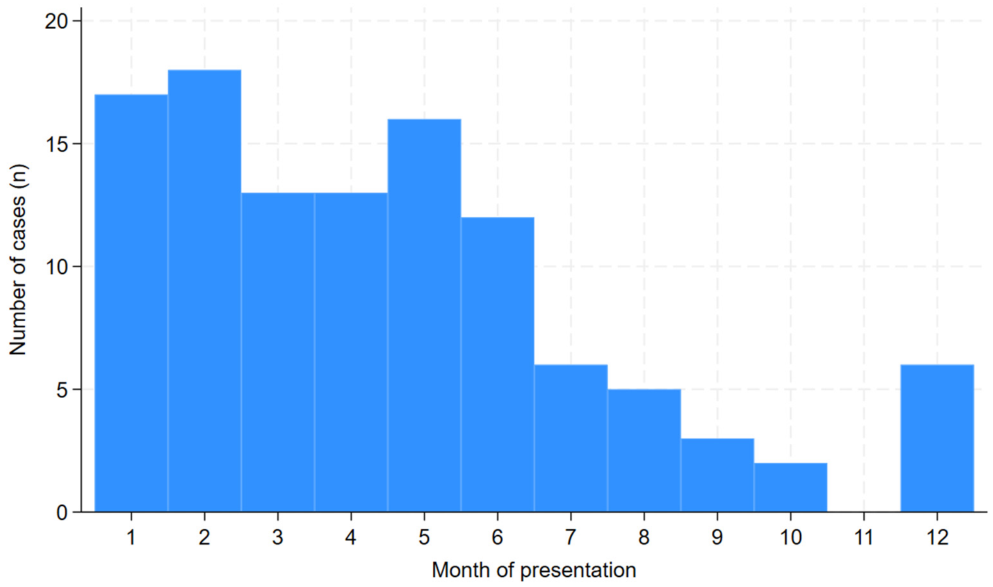

Supplementary Figure S2. Association between age and severe disease in the cohort

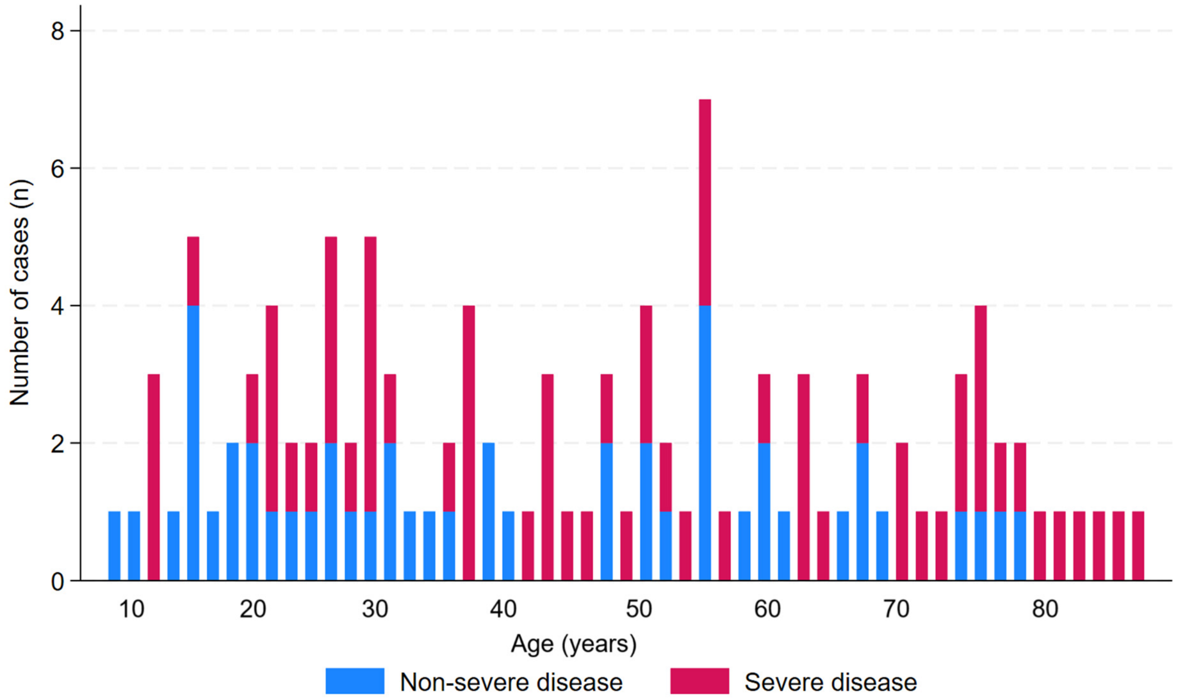

Supplement: Supplementary file 1 [file pathogens-14-00643-s001.zip › pathogens-3677001-supplementary.pdf]
